# News and Views (5&6): 50th Anniversary of IFM; A Panel Discussion at IUPAP100; Relaunch of Asian Network

**DOI:** 10.1007/s43673-023-00084-5

**Published:** 2023-05-22

**Authors:** AAPPS Bulletin

**Affiliations:** Association of Asia Pacific Physical Societies, Pohang, South Korea

## Institut Fizik Malaysia: the first fifty years by Bernardine R. Wong and Meng-Hock Koh

### Introduction

This year, 2023, marks a significant milestone in the history of physics in Malaysia. The Malaysian Institute of Physics (*Institut Fizik Malaysia*, IFM) was formed on January 29, 1973, and celebrates its golden jubilee this year. This article briefly traces the formation of IFM against the backdrop of physics in Malaysia, highlights some of the achievements and sketches possible future directions of IFM.

### Brief background of physics in Malaysia

A recent article [1] highlighted that the first X-ray photograph taken in present day Malaysia was made in 1897. If we consider that X-rays were discovered by Rontgen only 2 years earlier in 1895, it might suggest that the standard of physics teaching and research in British Malaya at that time was comparable to that of Europe. However, this would be misleading as the first X-ray apparatus installed at the Taiping Hospital (in present day Perak in Malaysia) on February 3, 1897, was presented in conjunction with the Diamond Jubilee celebration of Queen Victoria. At the time, this was the only hospital in the Far East to possess such an advanced diagnostic tool [2]. This prompted delivery of other X-ray devices for medical use in British Malaya over the following decades and specific training was given to technicians to handle the equipment [3]. However, a general post-secondary physics program only began to be offered in 1928, with the establishment of Raffles College in Singapore [4]. Eventually, Raffles College was merged with the King Edward VII College of Medicine (established in 1905) in 1949 to form, in Singapore, the University of Malaya. Subsequently, its Singapore campus became the National University of Singapore (NUS) while the Kuala Lumpur campus is *Universiti Malaya* (UM). Physics was offered as a degree programme in UM from 1961. Shortly thereafter, degree programs in physics began to be offered in *Institut Teknologi MARA* (ITM, established in 1967), *Universiti Sains Malaysia* (USM, 1969), *Universiti Kebangsaan Malaysia* (UKM, 1970), *Universiti Putra Malaysia* (UPM, 1971) and *Universiti Teknologi Malaysia* (UTM, 1972).

### Formation of IFM

As the number of physicists in Malaysia grew steadily in the early 1960s, a desire arose for a body to represent the physics community. Although some physicists in local universities were members of the Institute of Physics (United Kingdom), many physicists, teachers and those in the research and service sectors were not. This shortcoming began to be rectified when the first interim committee met in Kuala Lumpur in 1971 with the objective of setting up IFM. The institute’s constitution was drafted and approved by the Registrar of Societies, which enabled the process to proceed.

Written documentation preceding the formation of IFM is scarce; however, the IFM Constitution indicates the priorities that the framers had. These are (1) to promote physics and elevate the profession of physicists, (2) to educate and train those who wish to practice as physicists, and those who are interested in physics, (3) to hold meetings/conferences, workshops and to deliver lectures, and (4) to organize activities of interest to IFM members related to research, education, and outreach programs.

The inaugural general meeting of the IFM was held on January 29, 1973, at UM with 34 members. Apart from representation from *Universiti Malaya* (UM), *Universiti Sains Malaysia* (USM), *Universiti Kebangsaan Malaysia* (UKM), Agricultural College Serdang (now *Universiti Putra Malaysia*, UPM), Tunku Abdul Rahman College (TARC, now Tunku Abdul Rahman University of Management and Technology, TAR UMT) and Mara Institute of Technology (ITM, now *Universiti Institut Teknologi Mara* UITM), there was representation from the Meteorological Department, Defence Research Centre, Radioisotope Unit of *Universiti Hospital* (now University of Malaya Medical centre, PPUM), National Institute for Scientific and Industrial Research (NISIR), and Cochrane Road School. It is noteworthy that the diversity of the meeting attendees reflected the breadth of the impact of physics in the academic realm as well as in industrial research.

The inaugural IFM Council comprised Prof. Thong Saw Pak (president, TARC), Prof. Chatar Singh (vice-president, USM), Dr. Neo Yee Pan (hon. secretary, UM), Dr. Tan Hong Siang (hon. treasurer, UM), with the following council members: Dr. Khalijah Mohd. Salleh (UKM), Prof. Khaik Leang Lim (UM), Dr. Teh Hock Heng (UM), Dr. R. Ratnalingam (USM) and Dr. Lee Sing (UM). Altogether, there have been seven presidents: Prof. Thong Saw Pak (TARC, 1973–1975), Prof. Chatar Singh (1976–1977), Prof. Mohd. Zawawi Ismail (UKM, 1978), Prof. Tan Beng Cheok (UM, 1979–1990), Prof. Chia Swee Ping (UM, 1991–2010), Prof. Kurunathan Ratnavelu (UM, 2011–2020) and Prof. Tou Teck Yong (MMU, 2021–present). (Note: Prof. Lee Sing served as the acting president for some months in 1990.)

Since its inception, IFM has operated from the Department of Physics, UM. Starting with an initial 34 members in 1973, growth was slow but began to accelerate in the 1980s. To date, IFM has approximately 1530 members, of which 550 are active. IFM has nine Honorary Fellows and 54 Fellows. The IFM webpage is https://ifm.org.my/.

### Some IFM initiatives

IFM has co-organised a physics conference or symposium annually since 1975. It was initially known as the National Physics Symposium (NAPS) and is now more widely known as *Persidangan Fizik Kebangsaan* (PERFIK). Due to the Covid-19 pandemic, the most recent conferences, i.e., PERFIK 2021 and PERFIK 2022, were conducted online. It has been a valuable experience to have to rely solely on internet technologies. While it has been advantageous in widening participation, a purely online conference has come at a cost of a reduction in face-to-face interaction. Nevertheless, it is likely that some form of hybrid conference, workshop and meeting may become a new norm in the future.

Periodically, international events such as the International Conference on Frontiers in Quantum Physics (1997) [5], the International Meeting on Frontiers in Physics (IMFP 1998, 2005, 2009, 2013, 2017) [6, 7], and the Asia–Pacific Physics Conference (APPC, 1992 and 2019) [8, 9] have been held in Malaysia. In conjunction with APPC 2019, IFM also organised the Asia-Oceania Forum on Synchrotron Radiation Research, where representatives from synchrotron facilities in Australia, China, Japan, South Korea, Taiwan, Thailand, and Singapore shared the latest advances on the usage, research activities, and new instrumentation of synchrotrons. Recently, IFM collaborated with UCSI University to organize the 1st International Conference on Computational Science and Data Analytics (COMDATA 2021, 21–24 November, 2021).

IFM has also published the *Jurnal Fizik Malaysia* (JFM) since 1984, succeeding the *Bulletin of the Institute of Physics, Malaysia*, which was launched in 1980. Up until 2020, it appeared in print form but is now published solely online. *Jurnal Fizik Malaysia* is now indexed by Clarivate Analytics Emerging Sources Citation Index (ESCI). Information regarding submissions to JFM can be found at https://ifm.org.my/jurnal.

IFM has also supported the training of Malaysian high school students for the Asian and International Physics Olympiads (APhO and IPhO, respectively) since the 33rd IPhO in 2002 held in Bali, Indonesia. This has enabled our top physics students to compete with the best in the world. Following a preliminary test, selected students attend several intensive camps conducted by the trainers of *Universiti Kebangsaan Malaysia*, and by members of the IFM Education Subgroup. Five students are shortlisted to represent the country. Throughout the years of participation, Malaysian students have received honorable mention, bronze, silver, and gold medals. The students have benefited greatly from the exposure gained from the competition. Many have continued to pursue physics degrees and other science and engineering majors, and some have become prominent scientists and researchers in their own fields.

IFM has supported the training of young physicists in international schools via recommendations and partial financial assistance. Locally, IFM has initiated workshops and schools to train local and international physicists. Among the earliest training provided was a Workshop on Microcomputers and Applications (1981), in addition to other activities organised by IFM divisions and subgroups. IFM has two main divisions, i.e., Plasma Physics and Medical Physics. In addition to organising the Tropical College on Applied Physics (1983, 1986, 1988, 1992) [10–13], the plasma division has been active in initiating and strengthening plasma physics research in developing countries [14]. Recent activities include the Numerical Experiments Workshop on Plasma Focus (NEWPF2022), held over 7 weeks from 21 March–6 May 2022 by the plasma division, in collaboration with the Asian African Association for Plasma Training (AAAPT). The Medical Physics Division aims to promote medical physics [15]. Activities such as the Association of Southeast Asian Nations (ASEAN) College of Medical Physics (ACOMP) Courses 2021: Radiobiology in the era of precision medicine (9, 16, and 23 April 2021), International Medical Physics Week (9–13 May 2022), and the International Day of Medical Physics (November 7, 2022) involved online webinars that were very well received by the medical physics community. In addition, IFM has the following subgroups: Optics & Photonics, Theoretical & Computational Physics, Astronomy & Astrophysics, and the most recent subgroup is Industrial Physics, which was formed in 2021 to cater to the growing interest in the application of physics in industry. The Industrial Physics Subgroup has thus far organized two online seminars: The Application of Plasma Physics in Biomass Technologies, and What You Need to Know About Patents.

IFM is affiliated to the following organisations, as (1) a founding member of the Association of Asia Pacific Physical Societies (AAPPS), (2) a founding member of the Asia Pacific Center for Theoretical Physics (APCTP), (3) an associate council member of the Asia Oceania Forum for Synchrotron Radiation Research (AOFSRR), and (4) as a founding member of the Confederation of Scientific and Technological Associations in Malaysia (COSTAM). In addition, IFM cooperates with most other physics institutes/organizations of other countries.

### Looking forward

In its first 50 years, IFM established itself as a body supporting the interests of the local physics community through training, conferences, and the establishment of networks with the international physics communities. These can, and should, be continued. However, much remains to be done.

First, the physics community in Malaysia remains rather scattered with researchers typically working independently in their respective fields with minimal collaboration between them. Working in isolation robs researchers of the rewards of synergy that would then enable more impactful work to be attempted, such as those outlined in the Sustainable Development Goals (SDGs) of the United Nations [16]. IFM has a role to play to foster collaboration and, in so doing, to advance the status of physicists in the country. In the long run, we hope that IFM will be a point of reference for any governmental policies involving physics.

Second, IFM needs to better engage with the public to increase awareness of physics. Moving beyond how physics has traditionally been presented in a classroom setting, we need to use technology creatively to bring out the beauty of physics and the excitement of discovery that lies at its heart.

Third, there has been the perception that a physics degree does not appear to have a well-defined career path as opposed to, say, an engineering degree. In reality, a physics graduate is equipped with critical thinking skills and versatility so as to adapt to different career paths, whether in industry or the service sectors. IFM needs to initiate frequent engagement with industrial partners as well as more effectively organize outreach programs to schools.

Finally, researchers and students interested in exciting topics in fundamental physics, for example in particle physics, black holes, and search for dark matter, have difficulty securing positions in Malaysian universities, resulting in the brain drain of these talented researchers to other countries. It remains a challenge to build up a critical mass of physicists, and IFM has a role to play to address this issue.

**Fig. 1:**
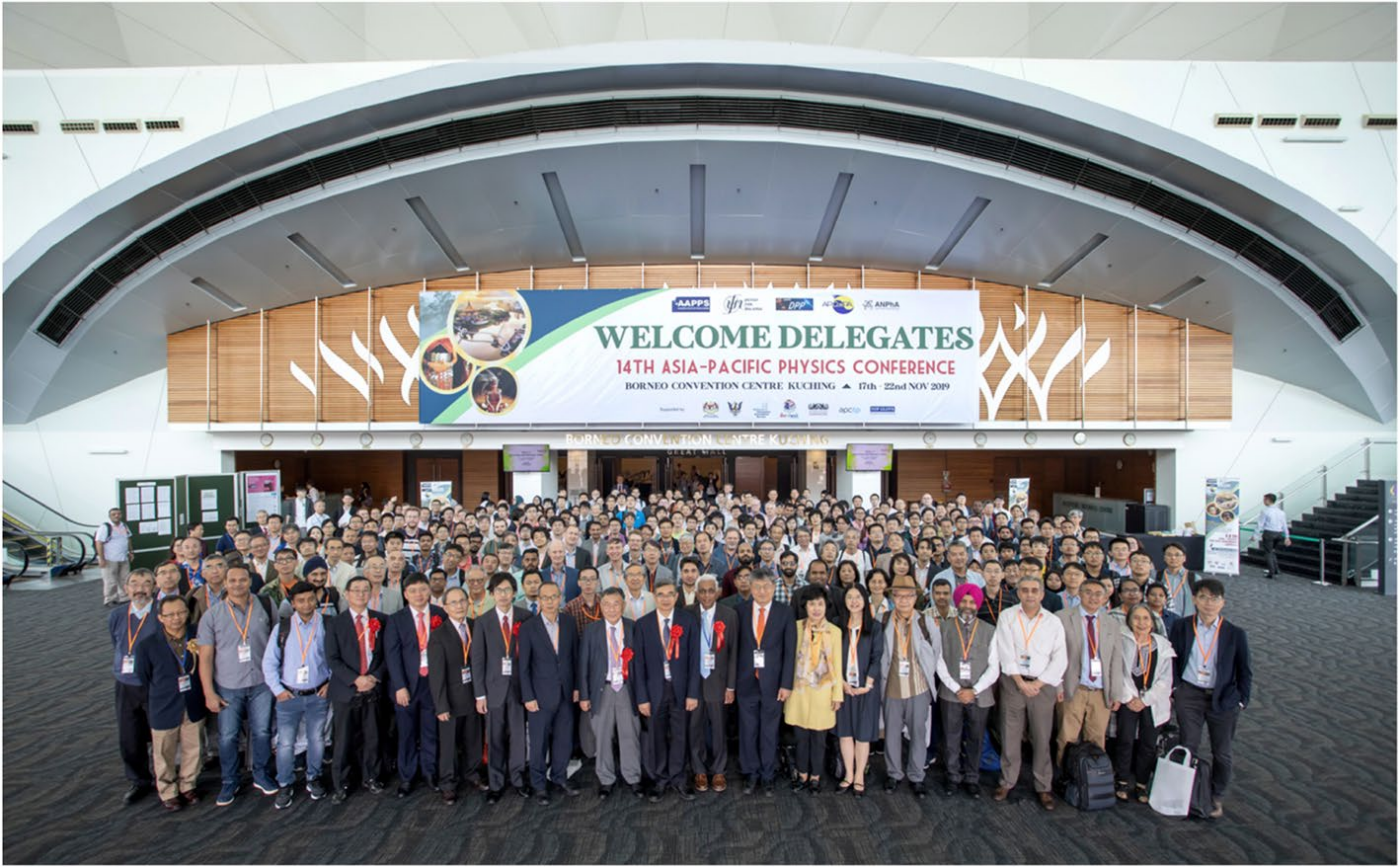
Participants of the 14th Asia–Pacific Physics Conference (APPC14) held in Kuching, Sarawak, Malaysia, from November 17–22, 2019. (Photograph courtesy of Prof. Kurunathan Ratnavelu.)

In conclusion, the first 50 years of IFM have been eventful both for the physics community as well as for our nation. Given the increased pace of change and advances in technology, we can expect IFM to rise to these challenges and continue to support the physics community in the years ahead. (Fig. 1).

**Acknowledgements**

We would like to express our thanks and appreciation to Prof. Tan Beng Cheok, Prof. Dato’ Roslan Abd. Shukor and Prof. Tou Teck Yong for their respective ideas and input in the preparation of this manuscript.

**Statements and declarations**

The authors declare that they have no competing interests.

## Panel on “Asia & Pacific: achievements in the region and expectations from IUPAP” by Kuijuan Jin, Mihoko Nojiri, Sunil Gupta, and Leong Chuan Kwek

**Fig. 2:**
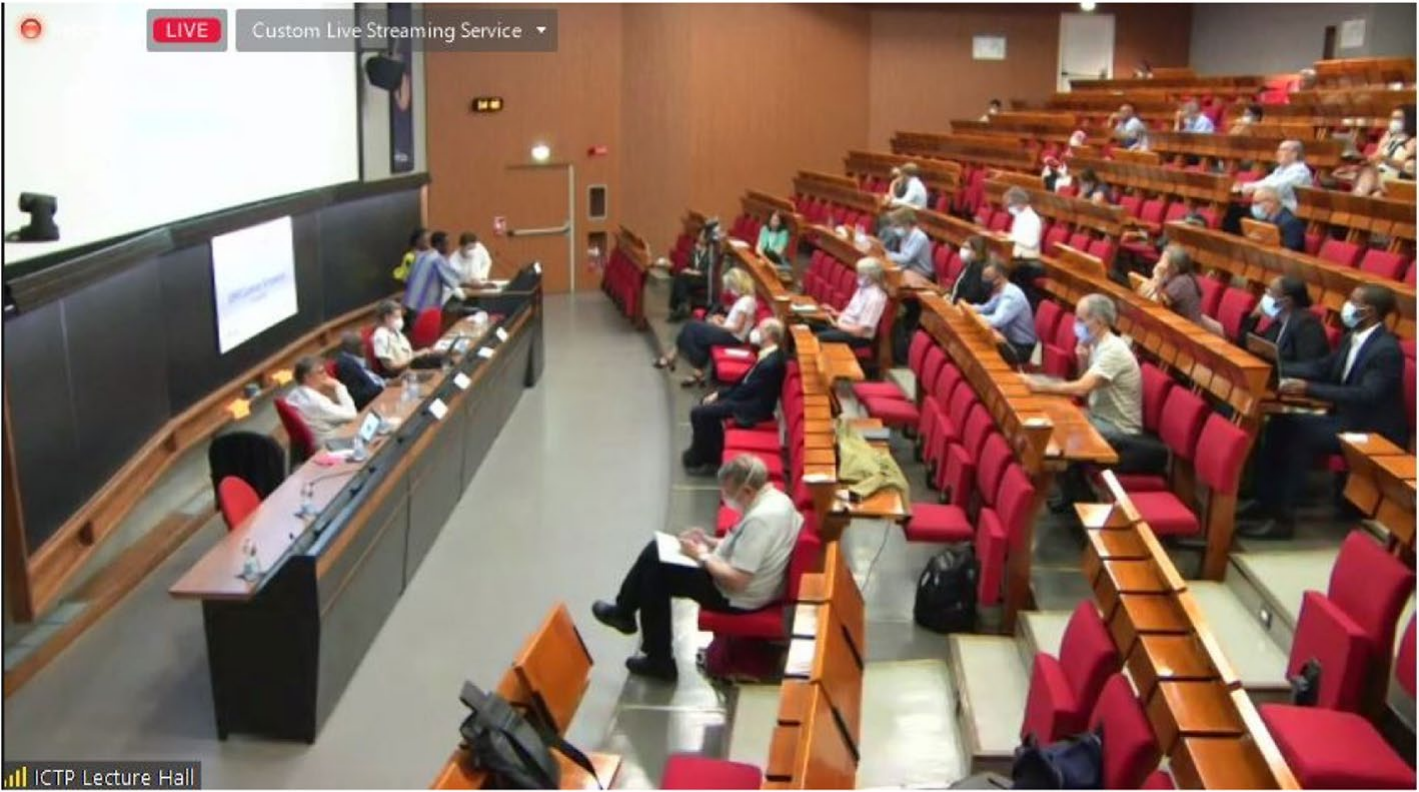
Hybrid session on 100th anniversary of the establishment of the International Union of Pure and Applied Physics

In conjunction with the 100th anniversary of the International Union of Pure and Applied Physics (IUPAP), members of Asia Pacific region organized a successful panel discussion at the main celebration event in Trieste on July 12, 2022. The panelists were William J. Munro (NTT, Japan), Xiaoyen Shen, Venu Gopal Achanta (Tata Institute of Fundamental Research), Junichi Yokoyama (President, AAPPS and University of Tokyo). Below are some of the key points that emerged from the Panel discussion in Trieste. (Fig. 2).

The discussion is restricted to just four topics which reflected major priority areas of Asia–Pacific region. But certainly the list was clearly not exhaustive by any means. To address that the following steps are token and below are some of the observations and recommendations,

- Mihoko had organized a virtual pre-Panel discussion lasting 90 min to give representation to the physical societies/regions that could not be accommodated in the Panel discussion in Trieste. We believe that the IUPAP100 has definitely made a big impact in the physics community in the AP region which needs to be sustained and harnessed through continued engagement and interaction through conferences/meetings.
- Given the reality of huge diversity in the level of physics education and research in this region, the support of the IUPAP EC would be absolutely critical in maintaining the momentum generated during IUPAP100.
- It has been observed that in several instances what the less developed regions lack in terms of physics infrastructure they make it up through strong commitment and drive. And therefore, a little help from the IUPAP would go a long way in sustaining and growing the physics infrastructure in those regions.
- There is also a varying degree of realization in many of the countries/regions that R&D and education in physics could be a major catalyst in the growth of their economies.
- Unfortunately, due to historical and geopolitical reasons the exchange of physicists or any collaboration among physicists in the AP region has been woefully short of what is required.

Therefore, the immediate recommendations are as follows:

- Utilize the momentum generated by IUPAP100 to develop an IUPAP (Asia–Pacific) website which is linked to the main IUPAP website. The current status is that the vendor for the development of IUPAP-AP site has been identified and approval of EC obtained for awarding contract. The partial payment to the web development company is being processed. Once that happens we would proceed with this activity.
- Foster close links with AAPS to better network physics community in AP region.
- Use the IUPAP-AP site to enable physicists in this region to organize virtual conferences with IUPAP sponsorship which obviate the need for travel which is very difficult for geopolitical and financial reasons.
- Encourage use of Indico to facilitate the hosting of these virtual conferences with emphasis on gender and geographical balance.
- Seek out the participation of eminent physicists from across the world by leveraging IUPAP sponsorship.
- Given the massive population and demographics the growth of physics in the AP region would be very important for the global development of physics.

## Relaunch of Asian Network on Condensed Matter, Complex Systems and Statistical Physics 2023–2026 by Paul A. Pearce and Sergej Flach

The Asian Network on Condensed Matter, Complex Systems and Statistical Physics (https://pcs.ibs.re.kr/ICTP_Asian_Network/ICTP_Asian_Network.html) was initially launched in 2017 and ran for the 3-year period 2017–2019. Unfortunately, due to COVID-19, the Network underwent a hiatus for 3 years from 2020 to 2022. We are now pleased to announce that, starting from 3 February, the Network is funded and relaunched to run for the 3-year period 2023–2026.

This theoretical physics Network is supported by funding from the International Center for Theoretical Physics (ICTP), the Asia Pacific Center for Theoretical Physics (APCTP), the Institute for Basic Science Center for Theoretical Physics of Complex Systems (PCS-IBS) and five participating Southeast Asian nodes in Vietnam, Thailand, Philippines, Indonesia, and Cambodia. Only the first three nodes were included in the first iteration of the Network and the Indonesian and Cambodian nodes were welcomed in the second expanded iteration of our Network. The ICTP and APCTP funding is provided through their various external activity programs. In principle, the ICTP funding can be extended for three more years but, ultimately, it is hoped that the Network will become self-sustaining with funding just from within Asia.

Notionally, a scientific Network consists of a number of Institutes and research groups in an entire region, pursuing a common scientific project over an extended period. The network program represents an efficient approach to overcoming the problem of isolation of scientists and stimulates interaction and collaboration. The Network contributes to regional efforts to advance scientific expertise in the developing world, providing young scientists in Southeast Asia with the further training and skills they need to enjoy productive careers. In effect, the combined expertise from ICTP, APCTP, and PCS-IBS is projected into Southeast Asia.

The activities of the Network include (i) scientist exchanges, (ii) an annual November School rotating between Hanoi, Nakhon Ratchasima and Manila, (iii) two annual workshops held at APCTP and PCS-IBS, and (iv) a Mini-School/Workshop to be held in Jakarta and Phnom Penh in 2024 and 2025 respectively. From 2017 through to 2019, the Network supported 12 scientist exchanges. Typically, a scientist visits more than one Institute in each supported itinerary of up to 4 weeks duration. It is expected that a similar number of scientist exchange visits will be supported between 2023 and 2026.

Over 150 scientists and many more graduate students are participating in our Network which benefits from the guidance and input from four distinguished International Scientific Advisers:

• Boris Altshuler, Columbia University, New York, USA
• Peter Fulde, Max Planck Institute, Dresden, Germany
• Giuseppe Mussardo, SISSA, Trieste, Italy

• Naoto Nagaosa, University of Tokyo, Tokyo, Japan

In addition, valuable expertise is gained from the participation of leading ICTP scientists including

• Marcello Dalmonte, CMSP Section
• Mikhail Kiselev, CMSP Section
• Antonello Scardicchio, CMSP Section
• Matteo Marsili, QLS Section

**Fig. 3:**
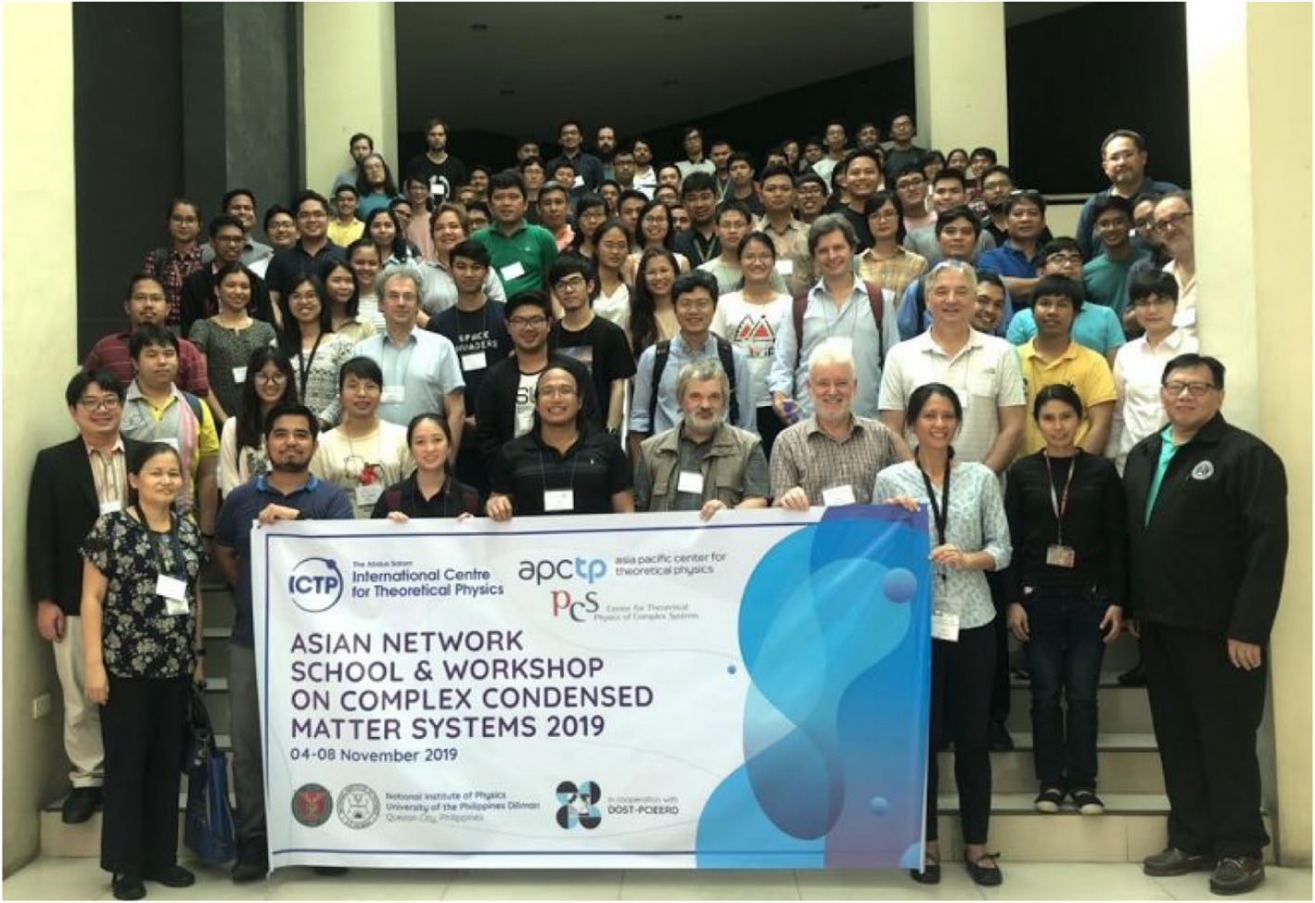
Group photo of our Network School held at NIP UP-Diliman, Manila, Philippines, 4–8 November 2019

The scientific program of this Network encompasses three disciplines with a focus on Condensed Matter Physics. Our aims are to cross-fertilize research on exciton–polariton condensates, superconducting networks, quantum dot networks, ultracold atomic gases, optical waveguide networks, topology, frustration, flatband physics, Fano resonant nanoscale devices, artificial gauge fields, dissipative quantum chaos, many body localization, quantum thermalization, quantum stochastic dynamics, targeted energy transfer, transport in nano structures, nonlinear nanophotonics, topological insulators, and more. In particular, within Condensed Matter Physics, we will focus on the following core topics of our research during the following years:

*• Complex condensed matter systems*
  Nonequilibrium many-body dynamics, macroscopic degeneracies, flat bands, non-Hermitian physics, optical cavities, and machine learning, with subtopics including exciton-polariton condensates, ultracold atomic gases, photonic waveguide networks, optical microcavities, Fano resonances, spin glasses, topology, frustration, disorder, many body localization, artificial gauge fields, dissipative quantum chaos, open quantum systems, quantum many-body interactions, nonlinear dynamics, disordered systems, mesoscopic electron transport, nano-electromechanical systems.
*• Light-matter interaction in nanostructures*
  Semiconductor microcavities, exciton polaritons, quantum transport, open quantum systems, quantum coherence, dissipative solitons, quantum dots, spins in mesostructures, polariton devices (signal routers, THz sources and detectors, lasers).
• *Strongly correlated electronic systems*
  Development of numerical algorithms to study correlated electronic systems, correlation effects in materials with strong spin–orbit coupling, computational study of spectral properties in strongly correlated systems, quantum embedding theories, dynamical mean field theory and impurity solvers.
  • Theoretical photonics: nonlinear optics, topological phases, non-Hermitian systems, disorder and Anderson localization, flat bands, scattering, Floquet systems, photonic lattices, solitons, and quantum optics.
*• Nonequilibrium quantum thermodynamics*
  Dissipative quantum systems, quantum and classical thermodynamics, quantum thermodynamic machines, nonlinear dynamics and thermodynamic phase transitions, heat transport in molecular junctions, open Floquet systems, symmetries and metastability in open systems, and Landau-Zener open systems.
*• Quantum chaos in many-body systems*
  Late-time quantum chaos and BGS conjecture, early-time quantum chaos and operator growth, quantum batteries, many-body localization, and quantum many-body scars.
*• Topological and correlated quantum matter*
  Topological and unconventional superconductivity with strong spin–orbit coupling, Moire materials and twistronics, bosonic topological phases in spin systems, nano-device applications of topological materials, aperiodic systems, and quasicrystals.
*• Optics of quantum fluids and nanomaterials*
  Optical properties of nanostructures, optical phonons in nanoparticles and Raman scattering, disorder effects on phonons in nanostructures, nonlinear aspects of lattice dynamics in nanoparticles, absorption and elastic scattering of light by nanodiamonds, quantum fluids of light, vortices and solitons dynamics, quantum turbulence, artificial photonic lattices, and effects of disorder on quantum fluid dynamics.
*• Superconducting hybrid quantum systems*
  Superconducting hybrid nanostructures, States read-out using circuit-QED, Majorana fermions in topological superconductors, magnetic impurities in superconductors, coherent electron transport in mesoscopic systems.
*• Entanglement and dynamics in quantum matter*
  Frustrated magnetism, quantum spin liquids, topological phases of matter, quantum dynamics, many-body localization, novel phases in ultra cold atoms, non-equilibrium quantum systems, quantum computing and algorithms, tensor networks, density matrix renormalization group, dynamical mean-field theory, and ab initio electronic structure computations.
  Although financial support from Network funds is limited to the members of the Network, all activities of the Network are open to participants from all ICTP supported (OEA) countries and all APCTP Member Countries. Lecturers that can support their own participation are particularly welcome at our Schools. (Fig. 3).

## References

[1] C.A.L. Lee, Physics Today **76**(2), 32 (2023). 10.1063/PT.3.5175

[2] History of Taiping Hospital, https://www.wikiwand.com/en/Taiping_Hospital#History . Accessed February 14, 2023

[3] A.A. Tajuddin, D.A. Bradley DA (eds.) *Centennial of the X-ray. An account of developments in radiological physics and radiology in Malaya and Malaysia* (Penang: Malaysian Institute of Physics, 1995)

[4] K.S. Lyn, *The Genesis of Higher Education in Colonial Malaya, Penang Institute*. (2018). https://www.ehm.my/publications/articles/the-genesis-of-higher-education-in-colonial-malaya. Accessed 14 Feb 2023

[5] S.C. Lim, R. Abd-Shukor, abd K.H. Kwek (eds.) *Proceedings of: International Conference on Frontiers in Quantum Physics, Kuala Lumpur, Malaysia, July 9–11, 1997* (Springer, Singapore, 1998)

[6] S.P. Chia, D.A. Bradley (eds.) *Proceedings of the International Meeting on Frontiers of Physics 1998: Kuala Lumpur, Malaysia, 26–29 October 1998* (World Scientific, Singapore, 2000)

[7] K. Ratnavelu, S.P. Chia, C.S. Wong, C.H. Ooi (eds.) International Meeting on Frontiers of Physics (IMFP) 2013, AIP. Conf. Proc. **1588**, 1 (2014). 10.1063/1.4866917

[8] S.P. Chia, K.S. Low, M. Othman, C.S. Wong, A.C. Chew, S.P. Moo (eds.) *Proceedings of the 5th Asia-Pacific Physics Conference, (2 volumes)*, (World Scientific, Singapore, 1992). 10.1142/2333

[9] Teck Yong Tou, Jun'ichi Yokoyama, Roslan Abdul Shukor, Kazuhiro Tanaka, Hyoung Joon Choi, Ryoji Matsumoto, Oi Hoong Chin, Jia Hou Chin and Kurunathan Ratnavelu, Proceedings of the 14th Asia-Pacific Physics Conference, AIP. Conf. Proc **2319** (2021). 10.1063/12.0003073

[10] S. Lee (ed.) *Proceedings of the F**irst Tropical College on Applied Physics, 26th December 1983 to 14th January, 1984* (World Scientific Pub., Singapore, 1985)

[11] S. Lee (ed.) *Proceedings of the Second Tropical College and Applied Physics: Laser and Plasma Technology, 17 March-5th April 1986, University of Malaya, Malaysia* (World Scientific Pub., Singapore, 1988)

[12] S. Lee, B.C. Tan, C.S. Wong, A.C. Chew, K.S. Low, S.P. Moo (eds.) *Proceedings of the Third Tropical College on Applied Physics* (World Scientific Pub., Singapore, 1990)

[13] B.C. Tan (ed.) *Proceedings of the Fourth Tropical College on Applied Physics: Laser and Plasma Technology, 28th May to 16th June 1990* (Narosa Pub., Kuala Lumpur, Malaysia, 1992)

[14] S. Lee and C.S. Wong, Physics Today **59**, 5, 31 (2006); 10.1063/1.2216959

[15] Jeannie Hsiu Ding Wong, Kwan Hoong Ng, Sivananthan Sarasanandarajah, Physica Medica **66**, 21-28 (2019). 10.1016/j.ejmp.2019.09.079 10.1016/j.ejmp.2019.09.079 31546154

[16] Transforming our world: the 2030 Agenda for Sustainable Development, https://sdgs.un.org/2030agenda . Accessed 16 Feb 2023

